# Does exercise participation promote happiness?: Mediations and heterogeneities

**DOI:** 10.3389/fpubh.2023.1033157

**Published:** 2023-03-10

**Authors:** Chao Li, Guangjie Ning, Yuxin Xia

**Affiliations:** ^1^Business School, Shandong University, Weihai, China; ^2^HSBC Business School, Peking University, Shenzhen, China

**Keywords:** happiness, health mechanism, demographic heterogeneities, mediations, exercise participation

## Abstract

This paper uses a nationally representative and large-scale dataset from China to empirically examine the relationship between exercise participation and happiness. To address the problem of reverse causality between the two factors, the instrumental variable (IV) approach is used to deal with endogeneity to some extent. It is demonstrated that higher frequencies of exercise participation are positively related to happiness. Findings also demonstrate that physical exercise could significantly decrease depressive disorders, improves self-rated health conditions and reduces the frequency of health problems affecting people's work and life. At the same time, all of above health factors significantly influence subjective wellbeing. When these health variables are included in regressions, the correlation between exercise participation and happiness declines. This confirms that physical activity helps to improve happiness by enhancing mental and overall health conditions. In addition, results show that physical activities are more prominently related to happiness for male, older and unmarried individuals and those living in rural areas, lacking social security and with higher levels of depression as well as lower socioeconomic status. Furthermore, a series of robustness checks are carried out and exercise participation's positive role in improving happiness is further confirmed using different happiness measures and instrumental variables, various IV models, as well as penalized machine learning methods and placebo tests. With the increasing emphasis of improving happiness as an important goal in the global public health policy, findings of this paper have important policy implications for enhancing subjective wellbeing.

## 1. Introduction

With the increase of people's willingness and ability to pursue a better life, happiness has not only received more and more attention from society, but also been recognized as an important goal in the global public policy (1). This concept appears in the literature in a variety of forms: subjective wellbeing, happiness, life satisfaction, general wellbeing, etc. These terms overlap to a large extent. Subjective wellbeing is widely used in the area of positive psychology and Diener et al. (2) give its classical definition from a hedonistic perspective. They believe that subjective wellbeing includes both cognitive assessment (i.e. overall life satisfaction) and affective wellbeing (comprising positive and negative emotions). This concept is further developed by introducing eudaimonic wellbeing, an aspect of wellbeing that consists of more than mere pleasure, but in the realization of one's human capital and true nature (3, 4). Happiness emphasizes individuals' overall evaluation of current life situation, and is widely considered as the most direct measure of people's subjective wellbeing (5).

In the past few decades, empirical research on subjective wellbeing has developed rapidly. Existing literature has studied different factors related to happiness from multiple aspects. First, income is the primary determinant of happiness, which was empirically confirmed in as early as 1984 (6). After that, most research has subsequently verified that income has significant impact on subjective wellbeing (7, 8). Besides, compared with the temporary change in earnings, income in people's life cycle has a greater impact on happiness (9, 10). Second, as for demographic and human capital characteristics, it has been shown a U-shaped relationship between age and subjective wellbeing, with people's happiness reaching its lowest level at midlife (11). In addition, better education is conducive to the improvement of subjective wellness (12). However, the impact of religious beliefs on happiness is not clear (13). Third, in terms of family characteristics, it is found that married individuals are generally happier (11), while the relationship between whether having children and subjective wellbeing is not statistically significant (6). Fourth, studies have also documented that job characteristics and work-related social security also affect happiness. For example, compared to informal employment, better social security that comes with formal employment contributes to the wellbeing of employees (14).

Furthermore, existing research suggests that physical fitness has an important impact on happiness, and there is also a significant association between exercise participation and health (15). So does exercise participation improve happiness? This is an important question yet to be rigorously tested. The frequency of physical activity has been shown to correlate with people's psychological states such as depression, anxiety, self-esteem, and wellbeing (16–19). Numerous studies have found a positive relationship between physical activity and happiness (20–26). Available evidence confirms that more frequent activity is associated with higher happiness levels (16, 27). In terms of the duration of physical activity, studies have found that even a short engagement in physical exercise is beneficial to happiness (28). With respect to the forms of activities, it is shown that both exercise and non-exercise have far-reaching benefits to physical health (29). Research has also proven that at least 10 min of physical exercise per week contributes to individuals' increased wellbeing (30). Lockdowns during COVID-19 increased the prevalence of anxiety and depressive disorders worldwide and immobility has been identified as one of the major causes behind these increases. (31). This appears to provide indirect evidence for the relationship between physical exercise and happiness. However, the relationship between exercise participation and happiness seems not to be a one-way direction because previous studies have also implied a bidirectional causality between the two factors. Happiness has an impact on people's physical activity and health behaviors, and consequently there is an interplay between happiness and physical exercise (32–34). Although research shows that the impact of happiness on physical activity is relatively weaker than that of physical activity on happiness (32, 35, 36), subjective wellbeing plays a role in affecting exercise participation (37). For example, individuals with higher happiness levels participate in exercise more frequently and suffer less from illness (38, 39). Moreover, a positive mental state helps to increase the probability of healthier behaviors and people with higher levels of happiness are more likely to maintain a healthy lifestyle (40).

Regarding how exercise participation affects happiness, first, it has been shown that physical fitness is one of the most important factors affecting people's wellbeing (41, 42). At the same time, other literature suggests that exercise is conducive to improving physical conditions (43, 44). Therefore, integrating the two streams of literature, it can be hypothesized that the positive effect of physical activities on happiness is exerted by improving people's physical health. Second, in terms of mental health, existing studies have detected that exercise helps to reduce psychological distress and decrease the risk of depression to some extent (45–47). Besides, available evidence confirms that there is a significantly negative correlation between happiness and depression (48, 49). Therefore, mental health status can also mediate the role of physical activity in impacting subjective wellbeing. Third, physical activity helps to increase the frequency of interpersonal interactions, thereby reducing social isolation (50). It has been shown that participation in physical activity can help people reduce loneliness and increase social participation (51–53). At the same time, loneliness is one of the important causes of people's poorer psychological state (54). A decrease in loneliness is significantly associated with an increase in wellbeing (55, 56). Therefore, physical exercise may affect wellbeing by influencing loneliness. Fourth, exercise can help people release stress. It has been found that regular physical activity makes people reduce their stress in life (57). Specifically, regardless of workplace stress for employees or academic stress for school students, exercise contributes to reducing these stresses (58–60). Furthermore, studies have shown a negative association between stress and wellbeing (61, 62). Thus, physical activity may affect wellbeing by influencing people's sense of stress. Fifth, from the perspective of lifestyle habits, physical activity may help people to maintain good habits, for example reducing sedentary behavior (63, 64). In addition, studies have also demonstrated a significant positive association between sedentary behavior and symptoms of depression and anxiety (65, 66). This implies that higher levels of sedentary behavior associate with decreased happiness (67). In a nutshell, from the available literature, it appears that exercise participation may influence happiness through mechanisms of physical and mental health, loneliness, stress, daily habits, etc. In addition, existing research suggests that there may exist heterogeneities in the relationship between exercise participation and happiness. For example, this correlation may vary by gender (39). Studies have shown that, among high school students, the relationship between exercise and happiness is more prominent in female than in male students (27). There are also variations among subgroups with different mental health conditions and physical activity is more positively associated with happiness for people with depression (68).

The objectives of this paper are threefold. First of all, this paper aims to clarify the relationship between exercise participation and happiness based on tackling endogeneity with the instrumental variable approach, applying a large-scale nationally representative data from China. Secondly, regarding how exercise may exert impacts on happiness, this research is to test how physical activity affects happiness using the mechanism analysis framework. Thirdly, this study also investigates the heterogeneities in the impact of physical exercise on subjective wellbeing. It is demonstrated that higher frequencies of exercise participation are positively related to happiness. Findings also demonstrate that physical exercise could significantly decrease depressive disorders, improves self-rated health conditions and reduces the frequency of health problems affecting people's work and life. At the same time, all of above health factors significantly influence subjective wellbeing. This confirms that physical activity helps to improve happiness by enhancing mental and overall health conditions. In addition, results show that physical activities are more prominently related to happiness for male, older and unmarried individuals and those living in rural areas, lacking social security and with higher levels of depression as well as lower socioeconomic status. Furthermore, a series of robustness checks are carried out and exercise participation's positive role in improving happiness is further confirmed using different happiness measures and instrumental variables, various IV models, as well as penalized machine learning methods and placebo tests.

Compared with the existing literature, contributions of this paper are mainly reflected in the following aspects. First, this paper deepens our understanding on the influencing factors of happiness from the perspective of exercise participation habits based on dealing with endogeneity. Previous studies have mainly focused on the effects of income (7–10), human capital characteristics (12, 13), family (6, 11) and working features (14) on subjective wellbeing. However, it can be concluded from above literature that the effect of exercise participation on happiness is unclear due to the possible bidirectional causality between the two factors. This paper deals with this issue by applying the instrumental variable approach to tackle endogeneity caused by reverse causality. Second, this paper clarifies how physical activity affects people's happiness. Regarding how exercise participation affects happiness, studies have shown that physical activity affects health as well as other factors, which are also associated with happiness. So whether physical exercise impacts happiness through these mechanisms need to be scientifically tested. This paper carries out an important exploration in this regard.

## 2. Materials, measures and methods

### 2.1. Data source

The dataset used in this paper is the Chinese General Social Survey (CGSS) collected in 2017 and 2018. CGSS is one of the most important nationally representative and large-scale academic survey projects in China, carried out by the national Survey Research Center at Renmin University of China (NSRC), which organizes the Chinese Social Survey Network (CSSN). The CGSS questionnaire aims to collect quantitative data about (1) measures of social structure, its stability and change, (2) measures of quality of life, objective and subjective, and (3) measures of underlying mechanisms linking social structure and quality of life. Detailed descriptions of CGSS are provided in the Supplementary material. CGSS has three advantages in this research. First, it directly investigates respondents' happiness level and contains comprehensive factors influencing happiness studied in the existing literature. This is conducive to examining the effects of exercise participation on happiness and conducting relevant mechanism and heterogeneity analysis. Second, CGSS covers information on people's exercise habits, facilitating the construction of explanatory variables. Third, CGSS contains detailed respondents' occupational codes of International Standard Classification of Occupations 2008 (ISCO-2008), which enable us to calculate the instrumental variable used in this paper to deal with endogeneity.

### 2.2. Measures

**Figure 1:**
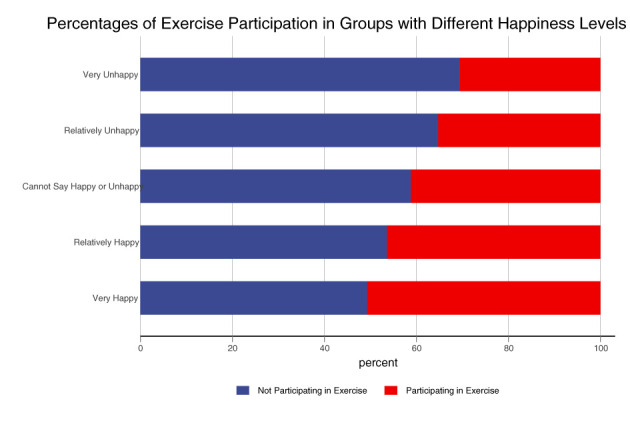
Relationship between exercise participation and happiness.

The main explained variable in this paper is the individual's happiness level, which is used in may existing studies (69–80). This measure is derived from the respondent's response to the question in CGSS: “Do you feel that you are generally happy in your life?” The answers to this question, based on the 5-Point Likert Scale, divide happiness levels into 1–5 levels, including very unhappy, relatively unhappy, cannot say happy or unhappy, relatively happy and very happy. In addition, CGSS has multidimensional subjective wellbeing indexes and the Cronbach's Alpha for the sub-scales is 0.891, demonstrating good internal consistency. The explanatory variable is the frequency of exercise participation used in Chen et al. (81), Lindwall and Hassmen (82), Wijngaards et al. (83), Anderson and Feldman (84), Gibney and Doyle (85), Brand et al. (86), Feng and Shi (87), Zhang and Zhang (88), Sun et al. (89), Wang and Wang (90), Wu (91). It comes from the question that “How many times a week do you engage in at least 30 min of physical activity that makes you sweat?”. By preliminarily analyzing the relationship between physical exercise and happiness, we find that in the very unhappy, relatively unhappy, cannot say happy or unhappy, relatively happy and very happy groups, the proportions of participating in physical exercise at least once a week are 30.508, 35.263, 41.194, 46.371, and 50.687%, respectively. Figure 1 illustrates that the larger the exercise participation rate, the higher the level of happiness. This implies a positive relationship between physical activity and happiness. In the following sections, we further examine the impact of physical exercise on happiness with more rigorous statistical analysis.

Based on the literature concerning the determinants of happiness (38, 92–94), to avoid omitted variable bias, this paper extensively controls factors influencing happiness in the following six aspects. (1) Demographic characteristics include age, the quadratic term of age and gender. (2) Human capital characteristics include education level and migration status. (3) Social characteristics include whether the respondent's Hukou^1^ is in urban, whether belonging to ethnic minorities, whether having religious beliefs and whether being the Communist Party of China (CPC) member. (4) Work characteristics include personal income, whether working in the system^2^ and whether having pension and medical insurance. (5) Family characteristics include whether married, family size, number of children and number of housing assets. (6) Regional and time characteristics include provincial and year dummies. The meanings and descriptive statistics of above variables are shown in Supplementary Table 1.

### 2.3. Methods

To examine the causal effect of exercise participation on happiness and to deal with the reverse causality problem, this paper uses the instrumental variable method to tackle endogeneity to some degree. Specifically, the following two-stage least squares (2SLS) statistical model is constructed.


$$Exercise_{i}=\alpha_{0}+\alpha_{1}Automation_{i}+x_{i}{}^{\prime }\psi^{1}+d_{y}+d_{p}+\varepsilon_{i}^{1}$$

$$Happiness_{i}=\beta_{0}+\beta_{1}\hat{Exercise_{i}}+x_{i}{}^{\prime }\psi^{2}+d_{y}+d_{p}+\varepsilon_{i}^{2}$$

In this model, *Exercise*_*i*_ and *Happiness*_*i*_ are exercise frequency and happiness of respondent *i*, respectively. The instrumental variable used in this paper is the degree to which automation affects the respondents, denoted as $Automation_{i}.\;x_{i}{}^{\prime }$ is a vector of control variables described above. *d*_*y*_ and *d*_*p*_ are time and provincial fixed effects. The first equation is the first stage regression of 2SLS, in which *Automation*_*i*_ is utilized to estimate *Exercise*_*i*_. In the second equation, the predicted values of exercise frequency from the first stage estimation are used to examine its effects on happiness. *Automation*_*i*_ is the instrumental variable constructed by Mihaylov and Tijden (95), which characterizes the degree of replacement by automation for occupations based on the routine intensity of the tasks at work. The automation index is calculated depending on respondents' occupation with Stata 17.0 MP, which is originally constructed by Mihaylov and Tijden (95). This measure is developed based on the task content of occupations under International Standard Classification of Occupations 2008 (ISCO-2008). Using a detailed set of 3,264 occupation-specific tasks, five sub-measures of non-routine analytic, non-routine interactive, routine cognitive, routine manual and non-routine manual tasks for 427 four-digit ISCO-2008 occupations are constructed. Details concerning this instrumental variable can be further referred to in Mihaylov and Tijden (95). Studies have shown that, the higher the degree of replacement by automation, the less time people need to work, and the more time they could spend on recreational activities and physical exercise (96). Therefore, this instrumental variable satisfies the correlation prerequisite. In addition, the impact of automation on occupation is determined by exogenous technological progress and thus is independent of individuals' characteristics. Consequently, this instrumental variable is exogenous to the estimation equation of individuals' happiness levels. Statistical test results of the correlation and exogeneity of this instrumental variable are presented in Supplementary Table 4. We also provided a detailed explanation and validity tests of the instrumental variable in the Supplementary material. Hence, applying the instrumental variable method, this paper can test the causal effect of exercise participation on happiness based on dealing with endogeneity to some extent. But it is important to note that the instrument variable approach often falls short of the “gold standard” of randomized controlled trials, because the assignment to the treatment of the instrument variable may not be totally random. Therefore, there may still exist endogeneity despite the use of this instrumental variable approach. All data processing and statistical analysis in this paper were conducted using Stata 17.0 MP - Parallel Edition.

## 3. Results

### 3.1. Benchmark results

Table 1 shows the regression results based on the above 2SLS model. Column (1) is the estimation that does not include any controls, demonstrating that exercise participation significantly positively impacts happiness. In columns (2–6), demographic characteristics, human capital characteristics, social characteristics, work characteristics, family characteristics and regional and time fixed effects are sequentially included in the regressions. In all the estimations, the coefficients of happiness are estimated to be significantly positive at the 1% level. This means that the more often people engage in physical exercise, the higher the level of their subjective wellbeing. In addition, with sequentially adding control variables in different aspects, the estimated coefficients of exercise participation are basically stable at around 0.290. This indicates that the significant correlation between physical activity and happiness is robust and not altered by other factors.

**Table 1:** Relationship between exercise participation and happiness.

**Model**	**(1) 2SLS**	**(2) 2SLS**	**(3) 2SLS**	**(4) 2SLS**	**(5) 2SLS**	**(6) 2SLS**
**Variable**	**Happiness**	**Happiness**	**Happiness**	**Happiness**	**Happiness**	**Happiness**
Exercise frequency	0.271^***^ (0.042)	0.255^***^ (0.043)	0.258^***^ (0.048)	0.335^***^ (0.092)	0.267^***^ (0.082)	0.290^***^ (0.109)
Age		−0.022^***^ (0.004)	−0.023^***^ (0.005)	−0.024^***^ (0.006)	−0.026^***^ (0.005)	−0.046^***^ (0.006)
Age_squared		0.000^***^ (0.000)	0.000^***^ (0.000)	0.000^***^ (0.000)	0.000^***^ (0.000)	0.000^***^ (0.000)
Whether female		0.079^***^ (0.022)	0.081^***^ (0.023)	0.097^***^ (0.029)	0.094^***^ (0.024)	0.085^***^ (0.026)
Education level			0.026 (0.041)	0.052 (0.039)	0.027 (0.034)	0.055 (0.038)
Whether migrants			−0.053^*^ (0.028)	−0.068^*^ (0.035)	−0.069^**^ (0.032)	−0.016 (0.034)
Whether Hukou in urban				−0.138^**^ (0.066)	−0.110^**^ (0.050)	−0.087 (0.053)
Whether ethnic minorities				0.173^***^ (0.059)	0.145^***^ (0.052)	0.176^***^ (0.055)
Whether religious believer				−0.054 (0.048)	−0.043 (0.045)	−0.050 (0.050)
Whether CPC member				0.004 (0.069)	0.033 (0.055)	−0.016 (0.066)
ln_Income					0.010 (0.006)	0.007 (0.007)
Whether working in the system					−0.002 (0.054)	−0.013 (0.067)
Whether having pension					0.108^***^ (0.026)	0.070^**^ (0.028)
Whether having medical insurance					0.053 (0.047)	0.025 (0.048)
Whether married						0.249^***^ (0.033)
Family size						0.023^***^ (0.009)
Number of children						0.041^**^ (0.018)
Number of houses						0.064^***^ (0.017)
Year dummies	No	No	No	No	No	Yes
Province dummies	No	No	No	No	No	Yes
Constant	3.296^***^ (0.086)	3.787^***^ (0.121)	3.803^***^ (0.117)	3.708^***^ (0.165)	3.670^***^ (0.139)	3.934^***^ (0.167)
Observations	12507	12507	12468	12436	11804	11725

^***^, ^**^, and ^*^Indicate significance at the levels of 1, 5, and 10%, respectively. The values in parentheses are standard errors robust to heteroskedasticity. Yes means the corresponding variables are controlled in the regression, while No means not controlled. 2SLS refers to the Two-Stage Least Squares model. Column (1) presents the estimation result of exercise participation on happiness without any control variables. Column (2) shows the estimation after adding the control variables of demographic characteristics. Column (3) further includes human capital and social identity characteristics. Column (4) further controls working characteristics. Column (5) further includes the family characteristics. Column (6) further controls regional and time dummies.

### 3.2. Mechanism analysis

The next question we care about is how exercise participation may enhance happiness. Based on existing literature, exercise participation may influence happiness through mechanisms of physical and mental health, loneliness, stress and daily habits. To tackle the problem of endogeneity, the 2SLS model is also used in the mechanism analysis based on the traditional three stage procedure, of which the details are provided in the Supplementary material. First, in terms of physical health, two indicators are utilized to characterize people's physical health status, including self-rated health status and the frequency of suffering from health problems. These mediators respectively come from respondents' evaluations on “how do you evaluate your current overall physical health status?”, and “how often do health problems affect your work and daily life?”. Based on the Likert scale, health status is classified into “very unhealthy”, “relatively unhealthy”, “fair”, “relatively healthy”, “very healthy” from 1-5. Besides, the values of the frequency of “suffering from health problems” are from 1 to 5 representing “never”, “seldom”, “sometimes”, “often” and “always” respectively. Column (2) of Table 2 indicates that physical exercise is positively correlated with the physical health status, while the estimate of exercise in column (4) is negatively correlated with the severity of health problems, which means that exercise participation could improve people's physical health. After the mediators are included in regressions, estimates of exercise participation in columns (3) and (5) decrease, meaning that the physical health plays a mediating role in the impact of physical activity on subjective wellbeing. This finding is consistent with existing studies (41–44). Second, as for the mental health, we use the measure of respondents' depression levels. It comes from answers on “how depressed do you feel?” Answers to this question are based on the Likert scale from 1 to 5, including “1-not depressed”, “2-mildly depressed”, “3-moderately depressed”, “4-very depressed” and “5-severely depressed” respectively. Results in column (6) show that physical exercise significantly reduces depression. Meanwhile, when both the explanatory and mediating variables are included in the regressions at the same time, as shown in column (7), the estimated coefficients of these two variables are significant. It means mental health also plays a mediating role in the impact of exercise frequency on happiness, consistent with previous research (33, 45–49, 97).

**Table 2:** Mechanism analysis.

**Model**	**(1) 2SLS**	**(2) 2SLS**	**(3) 2SLS**	**(4) 2SLS**	**(5) 2SLS**	**(6) 2SLS**	**(7) 2SLS**
**Variable**	**Happiness**	**Health status**	**Happiness**	**Suffering from health problems**	**Happiness**	**Depression**	**Happiness**
Exercise frequency	0.290^***^ (0.109)	0.466^***^ (0.159)	0.230^**^ (0.104)	−0.426^***^ (0.149)	0.257^**^ (0.111)	−0.328^**^ (0.129)	0.226^**^ (0.098)
Health status			0.133^***^ (0.024)				
Suffering from health problems					−0.097^***^ (0.023)		
Depression							−0.204^***^ (0.018)
Constant	3.934^***^ (0.167)	4.221^***^ (0.237)	3.369^***^ (0.134)	1.547^***^ (0.221)	4.085^***^ (0.174)	1.802^***^ (0.196)	4.297^***^ (0.160)
Controls	Yes	Yes	Yes	Yes	Yes	Yes	Yes
Observations	11725	11721	11721	11716	11716	11706	11706
**Model**	**(8) 2SLS**	**(9) 2SLS**	**(10) 2SLS**	**(11) 2SLS**	**(12) 2SLS**	**(13) 2SLS**	**(14) 2SLS**
**Variable**	**Happiness**	**Lonely_1**	**Happiness**	**Lonely_2**	**Happiness**	**Lonely_3**	**Happiness**
Exercise frequency	0.290^***^ (0.109)	0.185 (0.362)	0.427 (0.450)	−0.043 (0.222)	0.376 (0.391)	0.020 (0.227)	0.385 (0.396)
Lonely_1			−0.113 (0.083)				
Lonely_2					−0.156^***^ (0.056)		
Lonely_3							−0.135^*^ (0.072)
Constant	3.934^***^ (0.167)	4.221^***^ (0.237)	3.369^***^ (0.134)	1.547^***^ (0.221)	4.085^***^ (0.174)	1.802^***^ (0.196)	4.297^***^ (0.160)
Controls	Yes	Yes	Yes	Yes	Yes	Yes	Yes
Observations	11725	1956	1954	1956	1954	1956	1954

^***^, ^**^, and ^*^Indicate significance at the levels of 1, 5, and 10%, respectively. The values in parentheses are standard errors robust to heteroskedasticity. Yes means the corresponding variables are controlled in the regression, while No means not controlled. 2SLS refers to the Two-Stage Least Squares model. Columns (1) and (8) are the estimates using the instrumental variable approach in above analysis, demonstrating the effect of exercise frequency on happiness. Columns (2), (4), (6), (9), (11), and (13) present the estimations of exercise participation on mediating variables of physical health status, the frequency of suffering from health problems, depression, “Longly1”, “Lonely2” and “Lonely_3” respectively. Meanwhile, when both the explanatory and mediating variables are included in the regressions, corresponding results are shown in columns (3), (5), (7), (10), (12), and (14).

Third, we use respondents' ratings on the extent of “feeling lonely”, “feeling isolated by others” as well as “feeling lack of company” in CGSS to characterize their sense of loneliness, denoted as “lonely_1”, “lonely_2” and “lonely_3” respectively. Based on the Likert scale, ratings to these items are divided into “never”, “seldom”, “sometimes”, “often” and “always” from 1 to 5. When above mediators are included in the estimation of happiness, as demonstrated in columns (10), (12) and (14) of Table 2, both lonely_2 and lonely_3 are highly negatively correlated with happiness. This means that loneliness decreases happiness, which is consistent with existing literature. However, columns (9), (11), and (13) indicate that the estimated coefficients of exercise frequency on loneliness are not statistically significant. Therefore, loneliness does not play a mediating role in the impact of exercise on subjective wellbeing. This may stem from the fact that the physical activity indicator in this paper measures the frequency with which people participate in all types of exercise participation. Although it has been documented that group physical activities can help reduce loneliness (50–53), other types of exercises practiced by people themselves, such as running and swimming, do not necessarily play the same role. In addition, since without variables related to the respondents' sense of stress and other behavior habits in CGSS, we cannot conduct further mechanism analysis in this regard.

### 3.3. Heterogeneities analysis

This paper further investigates the heterogeneities of the effects of exercise participation on happiness among different subgroups with varied background characteristics, as shown in Table 3. In terms of gender, age and marital status, it is demonstrated that the positive relationship between physical activity and happiness is more pronounced for males, those over the age of 40 and unmarried individuals. This means that these groups tend to gain greater subjective wellbeing benefits from physical exercise. Moreover, we conduct a subsample analysis on people with different depression levels. Specifically, those who are very or severely depressed are treated as the more depressed group, and others are regarded as less depressed individuals. Results have demonstrated that the positive correlation between physical exercise and happiness tends to be much larger in those with higher levels of depression. This indicates that, for individuals with poorer mental health, exercise's role may be more effective in improving happiness.

**Table 3:** Heterogeneities analysis.

**Model**	**(1) 2SLS**	**(2) 2SLS**	**(3) 2SLS**	**(4) 2SLS**	**(5) 2SLS**	**(6) 2SLS**
**Sample**	**Male**	**Female**	**Younger than 40**	**Older than 40**	**Unmarried**	**Married**
**Variable**	**Happiness**	**Happiness**	**Happiness**	**Happiness**	**Happiness**	**Happiness**
Exercise frequency	0.292^**^ (0.133)	0.237 (0.151)	0.287 (0.213)	0.306^**^ (0.152)	0.331^*^ (0.197)	0.272^**^ (0.132)
Constant	3.629^***^ (0.246)	4.347^***^ (0.185)	2.935^***^ (0.378)	2.870^***^ (0.230)	3.503^***^ (0.539)	4.364^***^ (0.180)
Controls	Yes	Yes	Yes	Yes	Yes	Yes
Observations	6258	5467	4248	7477	2263	9462
**Model**	**(7) 2SLS**	**(8) 2SLS**	**(9) 2SLS**	**(10) 2SLS**	**(11) 2SLS**	**(12) 2SLS**
**Sample**	**Less depressed**	**More depressed**	**Not owing housing assets**	**Owning housing assets**	**Lower social status**	**Higher social status**
**Variable**	**Happiness**	**Happiness**	**Happiness**	**Happiness**	**Happiness**	**Happiness**
Exercise frequency	0.603 (0.793)	0.217^**^ (0.090)	0.285^*^ (0.170)	0.275^**^ (0.139)	0.325^*^ (0.171)	0.143 (0.119)
Constant	3.922^***^ (0.789)	3.754^***^ (0.171)	3.740^***^ (0.447)	4.188^***^ (0.202)	3.642^***^ (0.301)	4.346^***^ (0.175)
Controls	Yes	Yes	Yes	Yes	Yes	Yes
Observations	3303	8411	4326	7386	5875	5783
**Model**	**(13) 2SLS**	**(14) 2SLS**	**(15) 2SLS**	**(16) 2SLS**	**(17) 2SLS**	**(18) 2SLS**
**Sample**	**Lower social capital level**	**Higher social capital level**	**Living in rural areas**	**Living in urban areas**	**Having no pension**	**Having pension**
**Variable**	**Happiness**	**Happiness**	**Happiness**	**Happiness**	**Happiness**	**Happiness**
Exercise frequency	0.357^***^ (0.124)	2.327 (12.137)	0.424^**^ (0.188)	0.175 (0.197)	0.364^**^ (0.182)	0.252^**^ (0.127)
Constant	3.874^***^ (0.207)	1.757 (12.320)	3.778^***^ (0.328)	3.877^***^ (0.235)	4.041^***^ (0.337)	4.099^***^ (0.187)
Controls	Yes	Yes	Yes	Yes	Yes	Yes
Observations	9930	1771	8259	3466	3002	8723

^***^, ^**^, and ^*^Indicate significance at the levels of 1, 5, and 10%, respectively. The values in parentheses are standard errors robust to heteroskedasticity. Yes means the corresponding variables are controlled in the regression, while No means not controlled. 2SLS refers to the Two-Stage Least Squares model. Columns (1–18) are the estimated results for different subsamples shown in the “Sample” row.

Furthermore, we analyze the heterogeneities of exercise participation's effects on happiness from the perspectives of socioeconomic status. First, considering that housing property is an important aspect of economic assets, the heterogeneity analysis is carried out based on whether the respondent owns housing property. Results exhibit that the positive role of exercise participation tends to be more prominent for those not owning housing assets. This may be attributed to the fact that those owning housing property already have a higher level of happiness, so physical exercise's effects on improving their welfare are not pronounced. Mean values of the happiness measure for people owing housing property and their counterparts are 3.88 and 3.80 respectively. In addition, according to respondents' answers to the questions that “In general, are you in the middle and upper class in the society?” and “Can you take advantage of your current work to help your relatives and friends?”, we divide the sample into high and low social statuses and social capital groups. Subsample results indicate that the positive relationship between physical activities and happiness is more significant for those with lower social status and social capital level. Similarly, this result may also pertain to the fact that individuals with the higher social status and social capital level have higher wellbeing ratings, which are 4.08 and 3.99 respectively and much higher than 3.68 and 3.82 of their counterparts.

In terms of regional heterogeneity, results demonstrate that rural residents may be able to gain more benefits from exercise participation. This may be due to the better living standard in China's urban areas, resulting in a higher level of urban residents' happiness than rural respondents (3.95 > 3.81). Therefore, the benefits of physical exercise in improving happiness are not so obvious for urban participants. Meanwhile, in respect of social security, regardless of whether having pension, physical activity significantly promotes happiness. However, for those not having pension, the exercise participation's role is more prominent. Overall, the heterogeneity analysis suggests that physical activities are more prominently related to happiness for male, older and unmarried individuals and those living in rural areas, lacking social security and with higher levels of depression as well as lower socioeconomic status.

### 3.4. Robustness checks

This paper tests the robustness of the positive relationship between exercise participation and happiness in the following five ways. First, we conduct regressions using the dummy variable of happiness from the question of “whether feeling happy” as the new dependent variable applying the Probit model with the instrumental variable. As shown in Supplementary Table 2, the role of physical exercise is also significantly positive at the 1% level using this dependent variable, further confirming the robustness of the findings. Second, we use another measure of automation as the instrumental variable, constructed by Autor and Dorn (98). Results in Supplementary Table 3 demonstrate that the estimated coefficients using this instrumental variable are basically consistent with the benchmark results. Third, we perform robustness checks using other instrumental variable regression methods, including limited information maximum likelihood estimation (LIML) and generalized method of moments (GMM) models. As shown in Supplementary Table 4, no matter which instrumental variable estimation method is applied, the positive relationship between exercise participation and subjective wellbeing is robust. Fourth, we use penalized regressions to examine the predictive power of physical activity on happiness. Results of Supplementary Table 5 indicate that, exercise participation is an important indicator for predicting happiness in all of the penalized machine learning models, including Lasso, Ridge and Elastic Net. As illustrated in Supplementary Figures 1, 2, the predictive power of exercise participation on happiness is very robust with the increase of the penalty parameters. These results prove that exercise participation is a very important and robust factor to predict happiness compared to other variables. Fifth, a placebo test is performed on the relationship between exercise participation and happiness. Specifically, we randomly reassign the exercise participation variable 1000 times in the sample and perform regressions for the 1000 new samples. The distribution of exercise participation's estimated coefficients is illustrated in Supplementary Figure 3. The mean value of these 1000 estimates is close to 0, with more than 90% of their corresponding *P*-values > 0.1. This further demonstrates that the correlation between exercise participation and happiness cannot be attributed to other omitted random factors.

## 4. Discussion

On the basis of dealing with endogeneity problems including reverse causality to some degree, this paper confirms the positive relationship between exercise participation and happiness. This contributes to the existing literature where the causal relationship between the two factors is not systematically tested (32, 33, 39). Existing studies have explored the correlation between exercise and psychological state and subjective wellbeing, but it is difficult to investigate the causal relationship. For example, research based on the US data finds that regular exercise is negatively related to the frequency of depression and anxiety (17). In addition, a study on Norwegian adolescents detects that team exercise helps to reduce psychological distress and improve subjective wellbeing (27). In contrast to mental disorders, happiness is a kind of positive psychological state. Previous studies have shown that physical activities are positively associated with happiness, especially for individuals with long-term diseases (21) and the elderly (20, 24). Even leisure physical activities like outdoor walking are associated with higher scores on wellbeing (103, 104). Furthermore, in terms of the frequency of exercise, studies have found a stronger correlation between regular physical activity and happiness (28). However, past studies have suggested that happiness also affects people's exercise participation (32, 33). For example, a cohort study from Finland finds that people with higher levels of happiness are more likely to participate in sports activities (36). Therefore, it remains to be tested whether the positive correlation between exercise and wellbeing is a causal effect considering the problem of reverse causality. In other words, it is an open question whether exercise enhances subjective wellbeing. Based on dealing with the endogeneity problem caused by reverse causality using the instrumental variable approach to some extent, this paper proves that physical exercise can help to increases people's happiness level. Specifically, the more frequently people participate in physical activities, the higher the level of their subjective wellbeing. Besides, a series of robustness checks are conducted, which show that above findings are valid when using different happiness indicators, instrumental variables and IV models, as well as penalized machine learning methods and placebo tests.

In addition, this paper further identifies the reasons why exercise can enhance happiness. It has been shown that physical fitness is one of the most important factors affecting people's wellbeing (41, 42). At the same time, other literature suggests that physical activity is conducive to improving physical conditions (43, 44). Therefore, integrating the two streams of literature, the question naturally arises as to whether the positive effect of physical exercise on happiness is exerted by improving people's health. This paper supports the above hypothesis using the mechanism framework. Specifically, empirical results in this paper demonstrate that exercise participation improves subjective wellbeing by reducing depressive disorders, improving self-rated health status and decreasing the frequency of health problems affecting work and life. In addition, compared with previous studies that find positive effects of exercise (17, 27, 28, 103, 104), this paper further demonstrates the heterogeneities in the relationship between exercise participation and happiness across different subgroups. Specifically, this study shows that physical activities are more prominently related to happiness for male, older and unmarried individuals and those living in rural areas. Furthermore, exercise participation's role also varies among subgroups with different socioeconomic backgrounds and working characteristics. It is found that physical activity is more beneficial for people who lack social security and with lower socioeconomic status.

The shortcomings of this paper are mainly reflected in the following aspects. First, the Chinese General Social Survey used in this study, is a repeated cross-sectional dataset, which is limited to clarifying the bi-directional relationship between exercise participation and happiness. Although we use the instrumental variable approach to address the endogeneity problem, it still falls short of the “gold standard” of randomized controlled trials, because the assignment to the treatment of the instrument variable may not be totally random. The endogeneity of reverse causality needs to be better tackled based on an ideal prospective sample or randomized controlled trials. Second, since CGSS data is based on subjective answers, both the explanatory and the explained variables in this paper are subjective indicators. Although other kinds of variables are further used in the robustness checks, confirming the positive effect of exercise participation on happiness, the measures are also subjective indicators. Especially, for physical exercise, more scientific measures such as those in Riebe et al. (99), Perraton et al. (100), Morgan et al. (101), Stanton and Reaburn (102) need to be applied to test the findings of this paper. In addition, since without variables related to the respondents' sense of stress and other behavior habits in CGSS, we cannot conduct further mechanism analysis in this regard. Therefore, we look forward to further testing the relationship between physical exercise and happiness as well as the mechanisms based on better indicators in the future.

## 5. Conclusion

This paper empirically examines the relationship between exercise participation and happiness using data from the Chinese General Social Survey. Firstly, the endogeneity problem is tackled to some extent by applying the exogenous shock brought by automation as the instrumental variable to empirically investigate the causal effect. Analytical findings prove that physical exercise contributes to improving happiness. Secondly, it is found that exercise participation significantly decreases depression, improves self-rated health conditions and reduces the frequency of health problems affecting work and life, all of which help to promote happiness. Therefore, exercise participation could help to improve happiness by strengthening people's mental and overall self-rated health status. Thirdly, heterogeneities of the exercise participation's relationship with happiness are investigated. It is shown that for those who are male, older, unmarried, living in rural areas, lacking social security and with higher levels of depression as well as lower socioeconomic status, physical activity tends to play a more prominent role in enhancing happiness. Furthermore, the positive relationship between exercise participation and wellbeing are robust when using different happiness measures, instrumental variables and IV models, as well as penalized machine learning methods and placebo tests. This research has important implications for applying exercise participation to promote happiness. First, this paper proves the positive role of exercise participation in enhancing self-rated physical health, reducing depression levels and decreasing the sense of loneliness, thereby promoting subjective wellbeing. This implies that the health and subjective welfare benefits of exercise should be emphasized. Second, the heterogeneity analysis results have important implications. Those who are male, older, unmarried, living in rural areas, lacking social security and with higher levels of depression as well as lower socioeconomic status should be advised more to gain greater benefits from exercise participation.

## Data availability statement

The original contributions presented in the study are included in the article/Supplementary material, further inquiries can be directed to the corresponding authors.

## Ethics statement

The studies involving human participants were reviewed and approved by the Institutional Review Board, Renmin University of China. The patients/participants provided their written informed consent to participate in this study.

## Author contributions

CL contributed to the conception, design of the study, performed the statistical analysis, and wrote the first draft of the manuscript. YX generated the tables and figures respectively based on CL's analysis. GN worked on revisions of the manuscript. All authors provided critical feedback and approved the final submission.

## Footnotes

^1^Hukou is a system of household registration used in mainland China, mainly identifying a person as a rural or urban resident.

^2^Working in-system in China refers to having jobs in Communist Party of China organizations, governments and state-owned corporations. Compared with out-system jobs, in-system jobs bring better social security and additional hidden benefits.

## References

[1] Lancet T. Editorial: health and happiness. Lancet. (2016) 387:1251. 10.1016/S0140-6736(16)30062-9 27025416

[2] Diener E Suh EM Lucas RR Smith HL. Subjective well-being: three decades of progress. Psychol Bull. (1999) 125:276–302. 10.1037/0033-2909.125.2.276

[3] Ryan RM Deci EL. On happiness and human potentials: a review of research on hedonic and eudaimonic well-being. Annu Rev Psychol. (2001) 52:141–66. 10.1146/annurev.psych.52.1.141 11148302

[4] Deci EL Ryan RM. Hedonia, eudaimonia, and well-being: an introduction. J Happiness Stud. (2006) 9:1–11. 10.1007/s10902-006-9018-1

[5] Marttila E Koivula A Räsänen P. Does excessive social media use decrease subjective well-being? A longitudinal analysis of the relationship between problematic use, loneliness and life satisfaction. Telemat Inform. (2021) 59:101556. 10.1016/j.tele.2020.101556

[6] Diener E. Subjective well-being. Psychol Bull. (1984) 85:542–75. 10.1037/0033-2909.95.3.542 6399758

[7] McGuire J Kaiser C Bach-Mortensen AM. A systematic review and meta-analysis of the impact of cash transfers on subjective well-being and mental health in low-and middle-income countries. Nat Hum Behav. (2022) 6:359–70. 10.1038/s41562-021-01252-z 35058643

[8] Thomson RM Igelström E Purba AK Shimonovich M Pearce A Leyland A. How do changes in individual or household income impact on mental health for working-age adults? A systematic review. J Epidemiol Commun H. (2021) 75:71–71. 10.1136/jech-2021-SSMabstracts.152

[9] Cai S Park A. Permanent income and subjective well-being. J Econ Behav Organ. (2016) 130:298–319. 10.1016/j.jebo.2016.07.016

[10] Schöllgen I Kersten N Rose U. Income trajectories and subjective well-being: linking administrative records and survey data. Int J Env Res Pub He. (2019) 16:4779. 10.3390/ijerph16234779 31795266 PMC6926602

[11] Jebb AT Morrison M Tay L Diener E. Subjective well-being around the world: Trends and predictors across the life span. Psychol Sci. (2020) 31:293–305. 10.1177/0956797619898826 32045327

[12] Wang Z Sohail MT. Short-and long-run influence of education on subjective well-being: the role of information and communication technology in China. Front Psychol. (2022) 13:927562. 10.3389/fpsyg.2022.927562 35774959 PMC9237439

[13] Bian Y Zhang L Yang J Guo X Lei M. Subjective wellbeing of Chinese people: a multifaceted view. Soc Indic Res. (2015) 121:75–92. 10.1007/s11205-014-0626-6

[14] Morgan R O'Connor KJ. Labor market policy and subjective well-being during the great recession. J Happiness Stud. (2022) 23:391–422. 10.1007/s10902-021-00403-3

[15] Liu B Floud S Pirie K Green J Peto R Beral V. Does happiness itself directly affect mortality? The prospective UK million women. Study Lancet. (2016) 387:874–81. 10.1016/S0140-6736(15)01087-9 26684609 PMC5075047

[16] Ai X Yang J Lin Z Wan X. Mental health and the role of physical activity during the COVID-19 pandemic. Front Psychol. (2021) 12:1–8. 10.3389/fpsyg.2021.759987 34744938 PMC8565623

[17] Anderson E Shivakumar G. Effects of exercise and physical activity on anxiety. Front Psychiatry. (2013) 4:27. 10.3389/fpsyt.2013.00027 23630504 PMC3632802

[18] Giusti L Bianchini V Aggio A Mammarella S Salza A Necozione S. 12-month outcomes in overweight/obese users with mental disorders following a multi-element treatment including diet, physical activity, and positive thinking: the real-world ‘an apple a day' controlled trial. Front Psychiatry. (2022) 13:1–20. 10.3389/fpsyt.2022.903759 36081460 PMC9445251

[19] Bélair MA Kohen DE Kingsbury M Colman I. Relationship between leisure time physical activity, sedentary behaviour and symptoms of depression and anxiety: evidence from a population-based sample of Canadian adolescents. BMJ Open. (2018) 8:e021119. 10.1136/bmjopen-2017-021119 30337306 PMC6196847

[20] Felez-Nobrega M Haro JM Stubbs B Smith L Koyanagi A. Moving more, ageing happy: findings from six low-and middle-income countries. Age Ageing. (2021) 50:488–97. 10.1093/ageing/afaa137 32808968 PMC7936032

[21] Harvey C Ratcliffe P Gulliford MC. Well-being, physical activity and long-term conditions: cross-sectional analysis of Health Survey for England 2016. Public Health. (2020) 185:368–74. 10.1016/j.puhe.2020.06.013 32739777

[22] Gatab TA Pirhayti S. The effect of the selected exercise on male students' happiness and mental health. Procedia Soc Behav Sci. (2012) 46:2702–5. 10.1016/j.sbspro.2012.05.550

[23] Luo J Liu H Liu Y Jiang F Tang YL. Physical activity and mental health among physicians in tertiary psychiatric hospitals: a national crosssectional survey in China. Front Psychol. (2021) 12:731525. 10.3389/fpsyg.2021.731525 34721196 PMC8555760

[24] Khazaee-Pool M Sadeghi R Majlessi F Rahimi Foroushani A. Effects of physical exercise programme on happiness among older people. J Psychiatr Ment Health Nurs. (2015) 22:47–57. 10.1111/jpm.12168 25492721

[25] Castellanos-García P Lera-López F Sánchez-Santos JM. Light, moderate and vigorous physical activities: new insights into a virtuous circle with happiness. Eur J Sport Sci. (2022) 24:1–11. 10.1080/17461391.2022.2089053 35695097

[26] Ravari A Mirzaei T Bahremand R Raeisi M Kamiab Z. The effect of Pilates exercise on the happiness and depression of elderly women: a clinical trial study. J Sports Med Phys Fitness. (2020) 61:131–9. 10.23736/S0022-4707.20.10730-8 32734750

[27] Guddal MH Stensland SØ Småstuen MC Johnsen MB Zwart JA Storheim K. Physical activity and sport participation among adolescents: associations with mental health in different age groups. Results from the Young-HUNT study: a cross-sectional survey. BMJ Open. (2019) 9:e028555. 10.1136/bmjopen-2018-028555 31488476 PMC6731817

[28] Iwon K Skibinska J Jasielska D Kalwarczyk S. Elevating subjective well-being through physical exercises: an intervention study. Front Psychol. (2021) 12:702678 10.3389/fpsyg.2021.702678 34975608 PMC8719442

[29] Lathia N Sandstrom GM Mascolo C Rentfrow PJ. Happier people live more active lives: using smartphones to link happiness and physical activity. PLoS ONE. (2017) 12:e0160589. 10.1371/journal.pone.0160589 28052069 PMC5213770

[30] Zhang Z Chen W A. systematic review of the relationship between physical activity and happiness. J Happiness Stud. (2019) 20:1305–22. 10.1007/s10902-018-9976-0

[31] Lokman JC Bockting CL. Pathways to depressive and anxiety disorders during and after the COVID-19 pandemic. Lancet Psychiat. (2022) 9:531–3. 10.1016/S2215-0366(22)00152-3 35717953 PMC9212978

[32] Trudel-Fitzgerald C James P Kim ES Zevon ES Grodstein F Kubzansky LD. Prospective associations of happiness and optimism with lifestyle over up to two decades. Prev Med. (2019) 126:105754. 10.1016/j.ypmed.2019.105754 31220509 PMC6697576

[33] Saunders C Huta V Sweet SN. Physical activity, well-being, and the basic psychological needs: adopting the SDT model of eudaimonia in a post-cardiac rehabilitation sample. Appl Psychol Health Well Being. (2018) 10:347–67. 10.1111/aphw.12136 30027650

[34] Steptoe A Deaton A Stone AA. Subjective well-being, health, and ageing. Lancet. (2015) 385:640–8. 10.1016/S0140-6736(13)61489-0 25468152 PMC4339610

[35] Stenlund S Junttila N Koivumaa-Honkanen H Sillanmäki L Stenlund D Suominen S. Longitudinal stability and interrelations between health behavior and subjective well-being in a follow-up of nine years. PLoS ONE. (2021) 16:e0259280. 10.1371/journal.pone.0259280 34714864 PMC8555827

[36] Stenlund S Koivumaa-Honkanen H Sillanmäki L Lagström H Rautava P Suominen S. Changed health behavior improves subjective well-being and vice versa in a follow-up of 9 years. Health Qual Life Outcomes. (2022) 20:1–12. 10.1186/s12955-022-01972-4 35449057 PMC9027415

[37] Kubzansky LD Kim ES Salinas J Huffman JC Kawachi I. Happiness, health, and mortality. Lancet. (2016) 388:27. 10.1016/S0140-6736(16)30896-0 27397787

[38] Veenhoven R. Healthy happiness: Effects of happiness on physical health and the consequences for preventive health care. J Happiness Stud. (2008) 9:449–69. 10.1007/s10902-006-9042-1

[39] Kesavayuth D Shangkhum P Zikos V. Well-being and physical health: a mediation analysis. J Happiness Stud. (2022) 23:2849–79. 10.1007/s10902-022-00529-y

[40] Engels ES Reimers AK Pickel M Freund PA. Personality traits moderate the relationships between psychological needs and enjoyment of physical activity. Psychol Sport Exerc. (2022) 61:102197. 10.1016/j.psychsport.2022.10219733161044

[41] Lee M Yoon K. Effects of the health promotion programs on happiness. Sustainability. (2020) 12:528. 10.3390/su12020528

[42] Cobb S Javanbakht A Khalifeh Soltani E Bazargan M Assari S. Racial difference in the relationship between health and happiness in the United States. Psychol Res Behav Ma. (2020) 3:481–90. 10.2147/PRBM.S248633 32547270 PMC7259486

[43] Ao L Zhou J Han M Li H Li Y Pan Y. The joint effects of physical activity and air pollution on type 2 diabetes in older adults. BMC Geriatr. (2022) 22:1–11. 10.1186/s12877-022-03139-8 35650529 PMC9158242

[44] Zhao J Jiang W Wang X Cai Z Liu Z Liu G. Exercise, brain plasticity, and depression. CNS Neurosci Ther. (2020) 26:885–95. 10.1111/cns.13385 32491278 PMC7415205

[45] van Sluijs EMF Ekelund U Crochemore-Silva I Guthold R Ha A Lubans D. Physical activity behaviours in adolescence: current evidence and opportunities for intervention. Lancet. (2021) 398:429–42. 10.1016/S0140-6736(21)01259-9 34302767 PMC7612669

[46] Villani L Pastorino R Molinari E Anelli F Ricciardi W Graffigna G. Impact of the COVID-19 pandemic on psychological well-being of students in an Italian university: a web-based cross-sectional survey. Global Health. (2021) 17:1–14. 10.1186/s12992-021-00680-w 33823897 PMC8022300

[47] López-Bueno R Calatayud J Ezzatvar Y Casajús JA Smith L Andersen LL. Association between current physical activity and current perceived anxiety and mood in the initial phase of COVID-19 confinement. Front Psychiatry. (2020) 11:729. 10.3389/fpsyt.2020.00729 32793013 PMC7390883

[48] Mahmoodi Kahriz B Bower JL Glover FMGQ Vogt J. Wanting to be happy but not knowing how: poor attentional control and emotion-regulation abilities mediate the association between valuing happiness and depression. J Happiness Stud. (2019) 21:2583–601. 10.1007/s10902-019-00193-9

[49] Lin YH Chen HC Hsu NW Chou P. Validation of global self-rated health and happiness measures among older people in the Yilan study, Taiwan. Front Public Health. (2020) 8:1–9. 10.3389/fpubh.2020.00346 32850586 PMC7411153

[50] Rüth M Kaspar K. Educational and social exergaming: a perspective on physical, social, and educational benefits and pitfalls of exergaming at home during the covid-19 pandemic and afterwards. Front Psychol. (2021) 12:644036. 10.3389/fpsyg.2021.644036 33897546 PMC8062880

[51] Gyasi RM Phillips DR Asante F Boateng S. Physical activity and predictors of loneliness in community-dwelling older adults: the role of social connectedness. Geriatr Nurs. (2021) 42:592–8. 10.1016/j.gerinurse.2020.11.004 33246663

[52] Pinto A. de A, Oppong Asante K, Puga Barbosa RM, dos S, Nahas MV, Dias DT, Pelegrini A. Association between loneliness, physical activity, and participation in physical education among adolescents in Amazonas, Brazil. J Health Psychol. (2019) 26:650–8. 10.1177/1359105319833741 30841751

[53] Creese B Khan Z Henley W O'Dwyer S Corbett A Vasconcelos Da Silva M. Loneliness, physical activity, and mental health during COVID-19: a longitudinal analysis of depression and anxiety in adults over the age of 50 between 2015 and 2020. Int Psychogeriatr. (2020) 33:505–14. 10.1017/S1041610220004135 33327988 PMC7985900

[54] Sutton E Catling J Segaert K. Veldhuijzen van Zanten J. Cognitive health worries, reduced physical activity and fewer social interactions negatively impact psychological wellbeing in older adults during the COVID-19 pandemic. Front Psychol. (2022) 13: 823089. 10.3389/fpsyg.2022.823089 35250763 PMC8891508

[55] Stieger S Lewetz D Swami V. Emotional well-being under conditions of lockdown: an experience sampling study in Austria during the COVID-19 pandemic. J Happiness Stud. (2021) 22:2703–20. 10.1007/s10902-020-00337-2 33424431 PMC7778412

[56] Cauberghe V Van Wesenbeeck I De Jans S Hudders L Ponnet K. how adolescents use social media to cope with feelings of loneliness and anxiety during COVID-19 lockdown. Cyberpsych Beh Soc N. (2021) 24:250–7. 10.1089/cyber.2020.0478 33185488

[57] Vogel EA Zhang JS Peng K Heaney CA Lu Y Lounsbury D. Physical activity and stress management during COVID-19: a longitudinal survey study. Psychol Health. (2021) 37:51–61. 10.1080/08870446.2020.1869740 33405969

[58] Gerber M Schilling R Colledge F Ludyga S Pühse U Brand S. More than a simple pastime? The potential of physical activity to moderate the relationship between occupational stress and burnout symptoms. Int J Stress Manage. (2020) 27:53–64. 10.1037/str0000129

[59] Zhai X Ye M Wang C Gu Q Huang T Wang K. Associations among physical activity and smartphone use with perceived stress and sleep quality of Chinese college students. Ment Health Phys Act. (2020) 18:100323. 10.1016/j.mhpa.2020.100323

[60] Frömel K Šafár M Jakubec L Groffik D Žatka R. Academic stress and physical activity in adolescents. BioMed Res Int. (2020) 24:1–10. 10.1155/2020/4696592 32185205 PMC7060887

[61] Yarrington JS Lasser J Garcia D Vargas JH Couto DD Marafon T. Impact of the COVID-19 pandemic on mental health among 157,213 Americans. J Affect Disorders. (2021) 286:64–70. 10.1016/j.jad.2021.02.056 33677184 PMC9754791

[62] Mérida-López S Quintana-Orts C Rey L Extremera N. Teachers' subjective happiness: testing the importance of emotional intelligence facets beyond perceived stress. Psychol Res Behav Ma. (2022) 15:317–26. 10.2147/PRBM.S350191 35210880 PMC8859289

[63] Vandoni M Codella R Pippi R Carnevale Pellino V Lovecchio N Marin L. Combatting sedentary behaviors by delivering remote physical exercise in children and adolescents with obesity in the COVID-19 era: a narrative review. Nutrients. (2021) 13:4459. 10.3390/nu13124459 34960011 PMC8706684

[64] Hannan M Kringle E Hwang CL Laddu D. Behavioral medicine for sedentary behavior, daily physical activity, and exercise to prevent cardiovascular disease: a review. Curr Atheroscler Rep. (2021) 23:1–11. 10.1007/s11883-021-00948-x 34226989 PMC8257263

[65] Hallgren M Nguyen TTD Owen N Vancampfort D Smith L Dunstan DW. Associations of interruptions to leisure-time sedentary behaviour with symptoms of depression and anxiety. Transl Psychiat. (2020) 10:1–8. 10.1038/s41398-020-0810-1 32366824 PMC7198536

[66] Jiang L Cao Y Ni S Chen X Shen M Lv H. Association of sedentary behavior with anxiety, depression, and suicide ideation in college students. Front Psychiatry. (2020) 11: 566098. 10.3389/fpsyt.2020.566098 33424653 PMC7793895

[67] Felez-Nobrega M Olaya B Haro JM Stubbs B Smith L Koyanagi A. Associations between sedentary behavior and happiness: an analysis of influential factors among middle-aged and older adults from six low- and middle-income countries. Maturitas. (2021) 143:157–64. 10.1016/j.maturitas.2020.10.011 33308622

[68] Jiang W Luo J Guan H. Gender difference in the relationship of physical activity and subjective happiness among Chinese university students. Front Psychol. (2021) 12:1–6. 10.3389/fpsyg.2021.800515 34950093 PMC8688753

[69] Oshio T Nozaki K Kobayashi M. Relative income and happiness in Asia: Evidence from nationwide surveys in China, Japan, and Korea. Soc Indic Res. (2010) 104:351–67. 10.1007/s11205-010-9754-9

[70] Ha SE Jang SJ. National identity, national pride, and happiness: The case of South Korea. Soc Indic Res. (2014) 121:471–82. 10.1007/s11205-014-0641-7

[71] Lawrence EM Rogers RG Wadsworth T. Happiness and longevity in the United States. Soc Sci Med. (2015) 145:115–9. 10.1016/j.socscimed.2015.09.020 26421947 PMC4724393

[72] Wang BZ Cheng Z. Environmental perceptions, happiness and pro-environmental actions in China. Soc Indic Res. (2015) 132:357–75. 10.1007/s11205-015-1218-9

[73] Jackson J. Free to be happy: economic freedom and happiness in US states. J Happiness Stud. (2016) 18:1207–29. 10.1007/s10902-016-9770-9

[74] Okulicz-Kozaryn A Mazelis JM. More unequal in income, more unequal in wellbeing. Soc Indic Res. (2016) 132:953–75. 10.1007/s11205-016-1327-0

[75] Okulicz-Kozaryn A Golden L. Happiness is flextime. Appl Res Qual Life. (2017) 13:355–69. 10.1007/s11482-017-9525-8

[76] Sommet N. Elliot AJ. The effects of US county and state income inequality on self-reported happiness and health are equivalent to zero. Qual Life Res. (2022) 31:1999–2009. 10.1007/s11136-022-03137-8 35482148 PMC9188529

[77] Xu Q Sun W. Does financial inclusion promote investment and affect residents' happiness? —Evidence from China. Front Psychol. (2022) 13:988312. 10.3389/fpsyg.2022.988312 36072025 PMC9444132

[78] Su YS Lien D Yao Y. Economic growth and happiness in China: a Bayesian multilevel age-period-cohort analysis based on the CGSS data 2005–2015. Int Rev Econ Financ. (2022) 77:191–205. 10.1016/j.iref.2021.09.018

[79] Xu Z Ge R. The impact of energy consumption revolution on farmers' happiness: An empirical analysis from China. Front Public Health. (2022) 10:778002. 10.3389/fpubh.2022.778002 35356025 PMC8960032

[80] Zhang A Zhang Y Tao Y. Does retirement make people happier? -Evidence from China. Front Public Health. (2022) 10:874500. 10.3389/fpubh.2022.874500 35784195 PMC9247314

[81] Chen H Liu Y Zhu Z Li Z. Does where you live matter to your health? Investigating factors that influence the self-rated health of urban and rural Chinese residents: evidence drawn from Chinese general social survey data. Health Qual Life Outcomes. (2017) 15:78. 10.1186/s12955-017-0658-0 28431574 PMC5401560

[82] Lindwall M Hassmen P. The role of exercise and gender for physical self-perceptions and importance ratings in Swedish university students. Scand J Med Sci Sports. (2004) 14:373–80. 10.1046/j.1600-0838.2003.372.x 15546333

[83] Wijngaards I. del Pozo Cruz B, Gebel K, Ding D. Exercise frequency during the COVID-19 pandemic: A longitudinal probability survey of the US population. Prev Med Rep. (2022) 25:101680. 10.1016/j.pmedr.2021.101680 34976708 PMC8710431

[84] Anderson CL Feldman DB. Hope and physical exercise: the contributions of hope, self-efficacy, and optimism in accounting for variance in exercise frequency. Psychol Rep. (2019) 123:1145–59. 10.1177/0033294119851798 31142190

[85] Gibney S Doyle G. Self-rated health literacy is associated with exercise frequency among adults aged 50+ in Ireland. Eur J Public Health. (2017) 27:755–61. 10.1093/eurpub/ckx028 28371935

[86] Brand R Timme S Nosrat S. When pandemic hits: Exercise frequency and subjective well-being during COVID-19 pandemic. Front Psychol. (2020) 11:570567. 10.3389/fpsyg.2020.570567 33071902 PMC7541696

[87] Feng XS Shi P. Can the parental socio-economic status promote the children to participate in physical exercise? An empirical study based on the survey data of CGSS 2017. Eur Rev Med Pharmacol Sci. (2022) 26:4188-4296. 10.26355/eurrev_202206_29055 35776014

[88] Zhang S Zhang Y. The relationship between internet use and mental health among older adults in China: the mediating role of physical exercise. Risk Manag Healthc Policy. (2021) 14:4697–708. 10.2147/RMHP.S338183 34866945 PMC8633706

[89] Sun J Lyu S Dai Z. The impacts of socioeconomic status and lifestyle on health status of residents: Evidence from Chinese General Social Survey data. Int J Health Plann Mgmt. (2019) 34:1097–108. 10.1002/hpm.2760 30875102

[90] Wang Y Wang R. Impacts of physical exercise and media use on the physical and mental health of people with obesity: Based on the CGSS 2017 survey. Healthcare. (2022) 10:1740. 10.3390/healthcare10091740 36141352 PMC9498912

[91] Wu J. Exploring the mechanism analysis of men's retirement and physical activity participation based on the IV-Probit Model. Math Probl Eng. (2022) 2022:7409857. 10.1155/2022/7409857

[92] Bjegovic-Mikanovic V Wenzel H Laaser U. Data mining approach: what determines the well-being of women in Montenegro, North Macedonia, and Serbia? Front Public Health. (2022) 10:873845. 10.3389/fpubh.2022.873845 35719609 PMC9199491

[93] Song I Lee HJ. Predictors of subjective well-being in Korean men and women: analysis of nationwide panel survey data. PLoS ONE. (2022) 17:e0263170. 10.1371/journal.pone.0263170 35143526 PMC8830718

[94] D'Agostino A Grilli G Regoli A. The determinants of subjective well-being of young adults in Europe. Appl Res Qual Life. (2019) 14:85–112. 10.1007/s11482-017-9582-z28355635

[95] Mihaylov E Tijdens KG.Measuring the routine and non-routine task content of 427 four-digit ISCO-08 occupations. In: *Tinbergen Institute Discussion Paper*. Amsterdam: Tinbergen Institute (2019).

[96] Acemoglu D Restrepo P. Robots and jobs: evidence from US labor markets. J Polit Econ. (2020) 128:2188–244. 10.1086/705716

[97] Watson MC Lloyd J. Physical activity: manifold benefits for health and well-being. BMJ. (2022) 376:o815. 10.1136/bmj.o815 35354590

[98] Autor DH. David D. The growth of low-skill service jobs and the polarization of the US labor market. Am Econ Rev. (2013) 103:1553–97. 10.1257/aer.103.5.1553

[99] Riebe D Ehrman JK Liguori G Magal M. American College of Sports Medicine ACSM's Guidelines for Exercise Testing and Prescription. Philadelphia, PA: Lippincott Williams and Wilkins (2014).

[100] Perraton LG Kumar S Machotka Z. Exercise parameters in the treatment of clinical depression: a systematic review of randomized controlled trials. J Eval Clin Pract. (2010) 16:597–604. 10.1111/j.1365-2753.2009.01188.x 20039997

[101] Morgan JA Olagunju AT Corrigan F Baune BT. Does ceasing exercise induce depressive symptoms? A systematic review of experimental trials including immunological and neurogenic markers. J Affect Disord. (2018) 234:180–92. 10.1016/j.jad.2018.02.058 29529552

[102] Stanton R Reaburn P. Exercise and the treatment of depression: a review of the exercise program variables. J Sci Med Sport. (2014) 17:177–82. 10.1016/j.jsams.2013.03.010 23602562

[103] Rasciute S Downward P. Health or happiness? What is the impact of physical activity on the individual? Kyklos. (2010) 63:256–70. 10.1111/j.1467-6435.2010.00472.x

[104] Thaithatkul P Chalermpong S Laosinwattana W Kato H. Mobility, activities, and happiness in old age: case of the elderly in Bangkok. Case Stud Transp Policy. (2022) 10:1462–71. 10.1016/j.cstp.2022.05.010

