# Supplementary material for: Does exercise participation promote happiness?: Mediations and heterogeneities
Source: Front Public Health. 2023 Mar 10;11:1033157. doi: 10.3389/fpubh.2023.1033157 (PMC10036593; doi:10.3389/fpubh.2023.1033157)
Supplement: Supplementary file 1 [file Data_Sheet_1.pdf]

Supplementary Material

Supplementary Table 1. Variable Descriptions and Summary statistics

Variable	Description	Obs.	Mean	Std. Dev.	Min	Max
Dependent Variables						
Happiness	1-5 levels	12507	3.849	0.826	1	5
Whether happy	Yes=1, No=0	12507	0.778	0.415	0	1
Explanatory Variables						
Exercise participation		12507	2.037	3.078	0	21
Instrument Variables						
Automation	The degree of replacement by automation	12507	0.177	0.268	0	1
Control Variables						
Demographic Characteristics						
Age	Age	12507	45.959	13.694	18	101
Age squared	Squared term of age	12507	2299.718	1313.854	324	10201
Whether female	Yes=1, No=0	12507	0.469	0.499	0	1
Human Capital and Social Identities						
Education level	1-13 levels	12503	0.137	0.344	0	1
Whether migrant	Yes=1, No=0	12472	0.154	0.361	0	1
Social Characteristics						
Whether Hukou in urban	Yes=1, No=0	12482	0.299	0.458	0	1
Whether ethnic minorities	Yes=1, No=0	12507	0.081	0.374	0	1
Whether religious believer	Yes=1, No=0	12507	0.095	0.294	0	1
Whether CPC member	Yes=1, No=0	12492	0.102	0.303	0	1
Working Characteristics						
ln_Income	Logarithm of personal total income (RMB)	11943	9.414	2.792	0	16.113
Whether working in the system	Yes=1, No=0	12435	0.111	0.314		
Whether having pension	Yes=1, No=0	12490	0.743	0.437	0	1
Whether having medical insurance	Yes=1, No=0	12501	0.933	0.239	0	1
Family Characteristics						
Whether married	Yes=1, No=0	12507	0.802	0.398	0	1
Family size	Number of members in the family	12494	2.941	1.526	1	44
Number of children	Number of children in the family	12488	1.548	1.121	0	20
Number of houses	Number of houses in the family	12404	1.117	0.657	0	11
Year dummies						
Province Dummies						

Notes: The education level is classified from 1 to 13: 1-without any education, 2-kindergarten, 3-primary school, 4-junior high school, 5-vocational high school, 6-ordinary high school, 7-techinical secondary school, 8-technical high school, 9-junior college (adult education), 10-junior college (regular education), 11-undergraduate (adult education), 12-undergraduate (regular education), 13-postgraduate and above. The social contacts frequency is classified from 1 to 5: 1-never, 2-seldom, 3-sometimes, 4-often, 5-always. Hukou is a system of household registration used in mainland China, mainly identifying a person as a rural or urban resident. CPC is referred to Communist Party of China.

Supplementary Table 2. Robustness tests: using another happiness indicator

Model<br>Variable	(1)<br>IV-Probit<br>Whe_<br>happiness	(2)<br>IV-Probit<br>Whe_<br>happiness	(3)<br>IV-Probit<br>Whe_<br>happiness	(4)<br>IV-Probit<br>Whe_<br>happiness	(5)<br>IV-Probit<br>Whe_<br>happiness	(6)<br>IV-Probit<br>Whe_<br>happiness
Exercise participation	0.266***	0.256***	0.256***	0.277***	0.246***	0.277***
	(0.018)	(0.022)	(0.024)	(0.029)	(0.046)	(0.042)
Age		-0.017***	-0.017***	-0.016***	-0.021***	-0.040***
		(0.005)	(0.005)	(0.005)	(0.006)	(0.011)
Age_squared		0.000***	0.000***	0.000***	0.000***	0.000***
		(0.000)	(0.000)	(0.000)	(0.000)	(0.000)
Whether female		0.080***	0.082***	0.086***	0.097***	0.081***
		(0.022)	(0.022)	(0.021)	(0.024)	(0.024)
Education level			0.083	0.084*	0.063	0.088**
			(0.058)	(0.048)	(0.044)	(0.044)
Whether migrants			-0.095***	-0.093***	-0.108***	-0.041
			(0.032)	(0.031)	(0.035)	(0.040)
Whether Hukou in urban				-0.102**	-0.094**	-0.069
				(0.045)	(0.043)	(0.043)
Whether ethnic minorities				0.146***	0.143***	0.200***
				(0.040)	(0.044)	(0.062)
Whether religious believer				-0.044	-0.040	-0.048
				(0.040)	(0.045)	(0.046)
Whether CPC member				0.065	0.111	0.024
				(0.080)	(0.087)	(0.081)
ln_Income					0.010	0.006
					(0.008)	(0.008)
Whether working in the system					0.070	0.038
					(0.078)	(0.086)
Whether having pension					0.139***	0.089**
					(0.038)	(0.037)
Whether having medical insurance					0.055	0.016
					(0.049)	(0.047)
Whether married						0.244***
						(0.082)
Family size						0.027**
						(0.011)
Number of children						0.043***
						(0.014)
Number of houses						0.056*
						(0.031)
Year dummies	No	No	No	No	No	Yes
Province dummies	No	No	No	No	No	Yes
Constant	-0.044	0.355**	0.379**	0.265	0.295	0.475*
	(0.090)	(0.169)	(0.165)	(0.205)	(0.200)	(0.280)
Observations	12507	12507	12468	12436	11804	11725

Notes: ***, **, and * indicate significance at the levels of 1%, 5%, and 10%, respectively. The values in parentheses are standard errors robust to heteroskedasticity. Yes means the corresponding variables are controlled in the regression, while No means not controlled. IV-Probit refers to the Probit model with Instrument Variable. Column (1) presents the estimation result of exercise participation on happiness without any control variables.

Column (2) shows the estimation after adding the control variables of demographic characteristics. Column (3) further includes human capital and social identity characteristics. Column (4) further controls working characteristics. Column (5) further includes the family characteristics. Column (6) further controls regional and time dummies. The dummy variable of Whe_happiness comes from the question of "whether feeling happy or not", for which 1 denotes yes and 0 otherwise.

Supplementary Table 3. Robustness tests: using another instrumental variable

Model<br>Variable	(1) 2SLS<br>Happiness	(2) 2SLS<br>Happiness	(3) 2SLS<br>Happiness	(4) 2SLS<br>Happiness	(5) 2SLS<br>Happiness	(6) 2SLS<br>Happiness
Exercise participation	0.277***<br>(0.039)	0.281***<br>(0.042)	0.283***<br>(0.050)	0.337***<br>(0.078)	0.316***<br>(0.099)	0.358***<br>(0.121)
Age		-0.023***<br>(0.004)	-0.024***<br>(0.005)	-0.024***<br>(0.005)	-0.026***<br>(0.005)	-0.046***<br>(0.006)
Age_squared		0.000***<br>(0.000)	0.000***<br>(0.000)	0.000***<br>(0.000)	0.000***<br>(0.000)	0.000***<br>(0.000)
Whether female		0.087***<br>(0.023)	0.089***<br>(0.024)	0.099***<br>(0.028)	0.103***<br>(0.028)	0.095***<br>(0.030)
Education level			0.010<br>(0.043)	0.050<br>(0.038)	0.026<br>(0.037)	0.059<br>(0.043)
Whether migrants			-0.052*<br>(0.029)	-0.064*<br>(0.035)	-0.072**<br>(0.036)	-0.009<br>(0.038)
Whether Hukou in urban				-0.138**<br>(0.057)	-0.136**<br>(0.059)	-0.115*<br>(0.059)
Whether ethnic minorities				0.176***<br>(0.056)	0.167***<br>(0.059)	0.191***<br>(0.062)
Whether religious believer				-0.055<br>(0.047)	-0.057<br>(0.051)	-0.065<br>(0.056)
Whether CPC member				0.003<br>(0.062)	0.008<br>(0.064)	-0.051<br>(0.076)
ln_Income					0.007<br>(0.007)	0.003<br>(0.007)
Whether working in the system					-0.027<br>(0.064)	-0.048<br>(0.075)
Whether having pension					0.110***<br>(0.029)	0.072**<br>(0.032)
Whether having medical insurance					0.052<br>(0.051)	0.023<br>(0.054)
Whether married						0.245***<br>(0.037)
Family size						0.025**<br>(0.010)
Number of children						0.049**<br>(0.021)
Number of houses						0.060***<br>(0.020)
Year dummies	No	No	No	No	No	Yes
Province dummies	No	No	No	No	No	Yes
Constant	3.284***<br>(0.081)	3.735***<br>(0.127)	3.766***<br>(0.124)	3.700***<br>(0.156)	3.619***<br>(0.160)	3.870***<br>(0.191)
Observations	12558	12558	12519	12487	11855	11776

Notes: ***, **, and * indicate significance at the levels of 1%, 5%, and 10%, respectively. The values in parentheses are standard errors robust to heteroskedasticity. Yes means the corresponding variables are controlled in the regression, while No means not controlled. 2SLS refers to the Two-Stage Least Squares model. Column (1) presents the estimation result of exercise participation on happiness without any control variables. Column (2) shows the estimation after adding the control variables of demographic characteristics. Column (3) further includes human capital and social identity characteristics. Column (4) further controls working characteristics.

Column (5) further includes the family characteristics. Column (6) further controls regional and time dummies. The variable of Happiness is from the question in CGSS “Do you feel that you are generally happy in your life?”. The answers to this question measure happiness on a five-point Likert-scale with “1-very unhappy”, “2-relatively unhappy”, “3-cannot say happy or unhappy”, “4-relatively happy” and “5-very happy”.

Supplementary Table 4. Robustness tests: using different instrumental variable methods

Model / Variable	(1) First Stage Exercise frequency	(2) 2SLS Second Stage Happiness	(3) LIML Second Stage Happiness	(4) GMM Second Stage Happiness	(5) IGMM Second Stage Happiness
Exercise participation		0.290***	0.290***	0.290***	0.290***
		(0.109)	(0.109)	(0.109)	(0.109)
Routine tasks	0.388***				
	(0.117)				
Age	0.018	-0.046***	-0.046***	-0.046***	-0.046***
	(0.013)	(0.006)	(0.006)	(0.006)	(0.006)
Age_squared	-0.000	0.000***	0.000***	0.000***	0.000***
	(0.000)	(0.000)	(0.000)	(0.000)	(0.000)
Whether female	-0.152**	0.085***	0.085***	0.085***	0.085***
	(0.058)	(0.026)	(0.026)	(0.026)	(0.026)
Education level	-0.096	0.055	0.055	0.055	0.055
	(0.102)	(0.038)	(0.038)	(0.038)	(0.038)
Whether migrants	-0.046	-0.016	-0.016	-0.016	-0.016
	(0.091)	(0.034)	(0.034)	(0.034)	(0.034)
Whether Hukou in urban	0.380***	-0.087	-0.087	-0.087	-0.087
	(0.078)	(0.053)	(0.053)	(0.053)	(0.053)
Whether ethnic minorities	-0.199	0.176***	0.176***	0.176***	0.176***
	(0.136)	(0.055)	(0.055)	(0.055)	(0.055)
Whether religious believer	0.228*	-0.050	-0.050	-0.050	-0.050
	(0.116)	(0.050)	(0.050)	(0.050)	(0.050)
Whether CPC member	0.490***	-0.016	-0.016	-0.016	-0.016
	(0.111)	(0.066)	(0.066)	(0.066)	(0.066)
ln_Income	0.041***	0.007	0.007	0.007	0.007
	(0.011)	(0.007)	(0.007)	(0.007)	(0.007)
Whether working in the system	0.532***	-0.013	-0.013	-0.013	-0.013
	(0.101)	(0.067)	(0.067)	(0.067)	(0.067)
Whether having pension	-0.024	0.070**	0.070**	0.070**	0.070**
	(0.071)	(0.028)	(0.028)	(0.028)	(0.028)
Whether having medical insurance	0.036	0.025	0.025	0.025	0.025
	(0.120)	(0.048)	(0.048)	(0.048)	(0.048)
Whether married	0.043	0.249***	0.249***	0.249***	0.249***
	(0.079)	(0.033)	(0.033)	(0.033)	(0.033)
Family size	-0.014	0.023***	0.023***	0.023***	0.023***
	(0.020)	(0.009)	(0.009)	(0.009)	(0.009)
Number of children	-0.114***	0.041**	0.041**	0.041**	0.041**
	(0.032)	(0.018)	(0.018)	(0.018)	(0.018)
Number of houses	0.066	0.064***	0.064***	0.064***	0.064***
	(0.044)	(0.017)	(0.017)	(0.017)	(0.017)
Year dummies	Yes	Yes	Yes	Yes	Yes
Province dummies	Yes	Yes	Yes	Yes	Yes
Constant	0.754**	3.934***	3.934***	3.934***	3.934***
	(0.370)	(0.167)	(0.167)	(0.167)	(0.167)
Observations	11725	11725	11725	11725	11725

Notes: ***, **, and * indicate significance at the levels of 1%, 5%, and 10%, respectively. The values in parentheses are standard errors robust to heteroskedasticity. Yes means the corresponding variables are controlled in the regression, while No means not controlled. 2SLS refers to the Two-Stage Least Squares model. LIML refers to Limited Information Maximum Likelihood Estimation. GMM refers to Generalized Method of Moments. IGMM refers to Iterative Generalized Method of Moments. The variable of Happiness is from the question in CGSS "Do you feel that you are generally happy in your life?". The answers to this question measure happiness on a five-point Likert-scale with "1-very unhappy", "2-relatively unhappy", "3-cannot say happy or unhappy", "4-relatively happy" and "5-very happy".

Supplementary Table 5. Robustness tests: using panelized machine learning methods

Model<br><br><br>Variable	(1)<br>Lasso<br>(10-fold CV)<br>Happiness	(2)<br>Lasso<br>(20-fold CV)<br>Happiness	(3)<br>Ridge<br>(10-fold CV)<br>Happiness	(4)<br>Ridge<br>(20-fold CV)<br>Happiness	(5)<br>Elastic Net<br>(10-fold CV)<br>Happiness	(6)<br>Elastic Net<br>(20-fold CV)<br>Happiness
Exercise participation	0.0250	0.0250	0.0245	0.0245	0.0250	0.0250
No. of nonzero coefficients	46	46	48	48	46	46
λ	0.0002	0.0003	0.0093	0.0093	0.0002	0.0003
Out-of-sample R^2	0.0710	0.0707	0.0682	0.0679	0.0710	0.0707
CV mean prediction error	0.6457	0.6459	0.6476	0.6478	0.6457	0.6459
α					1	1
Observations	23345	23345	23345	23345	23345	23345

Notes: ***, **, and * indicate significance at the levels of 1%, 5%, and 10%, respectively. The values in parentheses are standard errors robust to heteroskedasticity. Yes means the corresponding variables are controlled in the regression, while No means not controlled. Lasso refers to Least Absolute Shrinkage and Selection Operator. CV refers to cross-validation. The variable of Happiness is from the question in CGSS "Do you feel that you are generally happy in your life?". The answers to this question measure happiness on a five-point Likert-scale with "1-very unhappy", "2-relatively unhappy", "3-cannot say happy or unhappy", "4-relatively happy" and "5-very happy". The number of independent predictors included in the Penalized Machine Learning models is 51.

Supplementary Figure 1. Coefficient Paths in the Lasso/Elastic Net Model

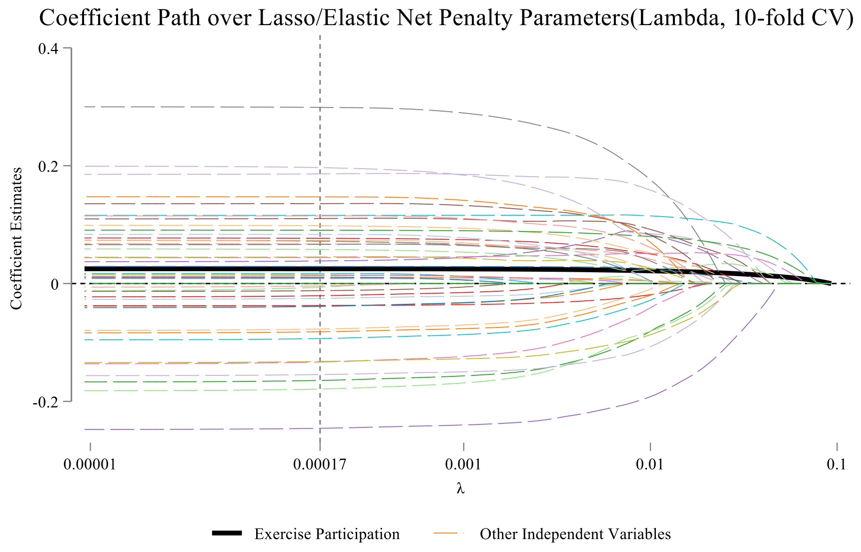

Supplementary Figure 2. Coefficient Paths in the Ridge Model

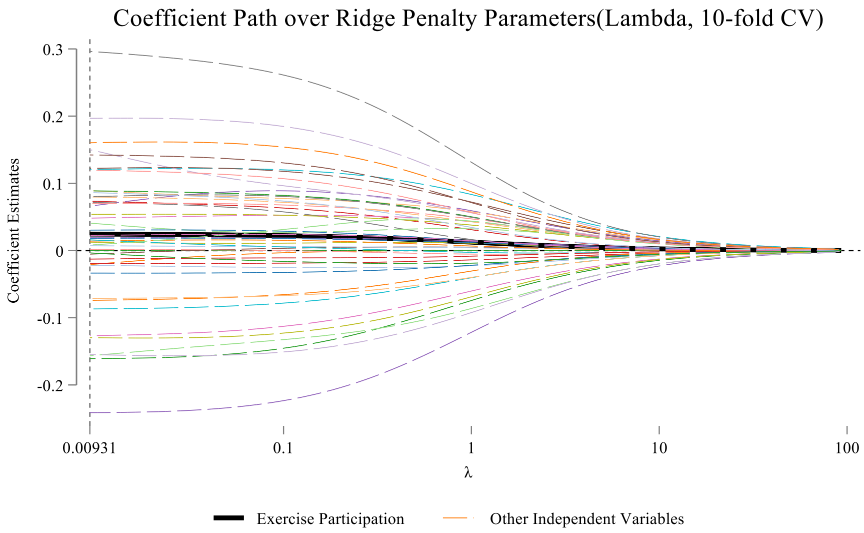

α_{CV} Cross-validation minimum alpha. α=0

Supplementary Figure 3. Placebo Test

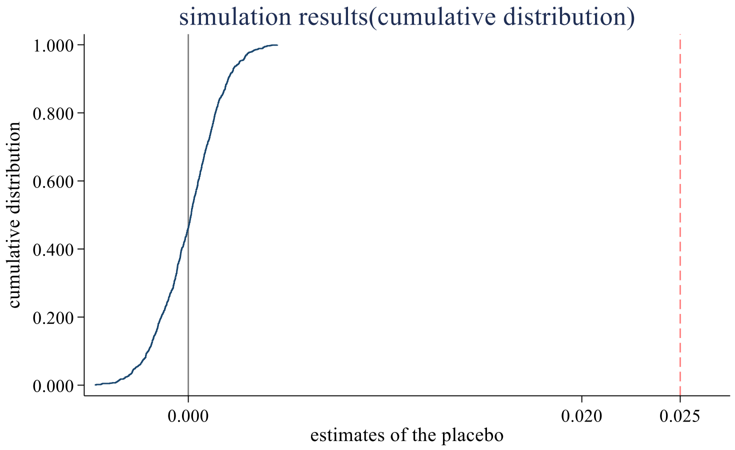

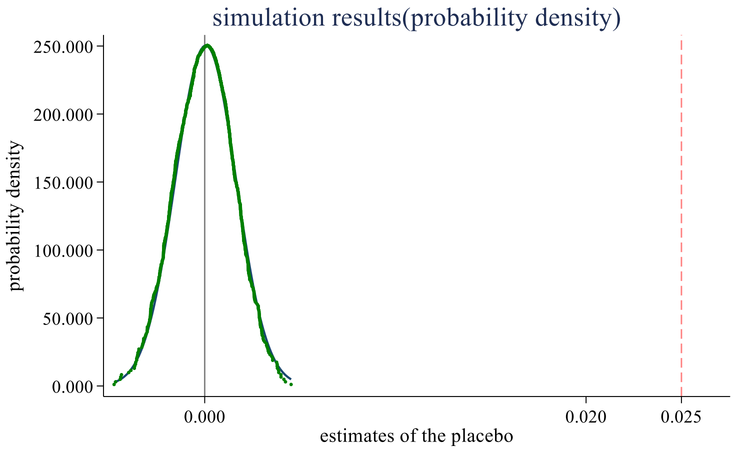

Supplementary Introduction to the Dataset

This paper uses a large-scale and nationally representative dataset, which is the Chinese General Social Survey (CGSS). CGSS is in the world General Social Survey family, carried out by the national Survey Research Center at Renmin University of China (NSRC), which organizes the Chinese Social Survey Network (CSSN) including 49 universities and provincial social science academies. The CGSS aims to collect quantitative data about (1) measures of social structure, its stability and change, (2) measures of quality of life, objective and subjective, and (3) measures of underlying mechanisms linking social structure and quality of life. The questionnaire of CGSS composes three kinds of modules: core module, topic module (rotation module), and additional module. The core module is annually repeated, which includes 152 variables. There are two kinds of the CGSS core module variables. One is the standard background variables, which include 71 variables. Another is the variables to trace social change trends. They are the rest 81 variables. The core module of the CGSS has 11 dimensions: Social demographic, health, lifestyle, migration, social attitude, class identity, political attitude and behavior, cognitive ability, labor market participation, social welfare, and family. The topic modules will be rotated every five years. There are one or two topic modules in the annual questionnaire. The topic modules aim to address important social issues. The additional modules include EASS module, ISSP module, and other ad hoc one-time modules. Some proposed topic modules also might be used as one-time additional modules. Questions in core module and topic modules will be asked to all respondents. Questions in additional modules only have one-third or a half chance to be asked. The questionnaire of CGSS can be accessed through http://cgss.ruc.edu.cn/English/Home.htm and http://www.cnsda.org/.

The sampling of CGSS is based on the multi-stage stratified design. The sampling stages are as follows: (1) PSUs are county-level units and there are 2762 PSUs in the sampling frame; (2) SSUs are community-level units (villages (cun] and neighborhood committees (ju wei hui]); (3) in selected SSU, 25 households (TSUs) are sampled with PPS method; (4) one eligible person aged 18-above is selected from each sampled household to serve as the representative. There are 43 Municipalities directly under the Chinese central government, provincial capital cities, and vice provincial cities in China. Comprehensive ranking by GDP, FDI and Education Level to these cities, the top 5 is Beijing, Shanghai, Tianjin, Guangzhou, and Shenzhen. CGSS treats these 5 cities as self-representative stratum. This stratum consists of 67 PSUs. The rest 2695 PSUs are comprehensively ranked with GDP per capital, urbanization rate, and population density and then are equally classified into 50

strata. Within each stratum, 2 PSUs will be selected with PPS method. In each selected PSU, 4 communities are sampled with PPS method. There are 80 communities in self-representative stratum and 400 communities in the rest 50 strata.

The national Survey Research Center at Renmin University of China (NSRC) organized Chinese Social Survey Network (CSSN), including 49 universities and provincial social science academies. Members of CSSN undertake the survey of the CGSS in their own provinces. The CGSS uses Computer Aided Personal Interviewing, and the average interview time is about one and a half hours. The CGSS has a set of strict quality control procedures, which cover pre-fieldwork, in-fieldwork, and after-fieldwork states. In pre-fieldwork stage, all supervisors must receive 40 hours training and finish 4 experimental interviews; all interviewers must receive 25 hours training and finish 3 experimental interviews. In in-fieldwork stage, all interviewers will be accompanied to interview by supervisor at least once. And the finished questionnaires must be 100% on site check and supervisors must do 40% the second day back interviewing. And in post-fieldwork stage, all interviews must 100% mail back interview and 40% telephone interview. In data input and coding stage, the data must be double input and double coding and there are several supervisors to check the double input and coding validation process. Details of the data collection process can be accessed through http://cgss.ruc.edu.cn/English/Home.htm.

Above information is taken from http://cgss.ruc.edu.cn/English/Home.htm.

Explanation and Validity Tests of The Instrumental Variable

The Chinese General Social Survey (CGSS), which is used in this study, is a repeated cross-sectional dataset, rather than prospective, longitudinal or panel data. If we use the simple Ordinary Least Squares (OLS) model to perform the following statistical analysis with cross-sectional data, the estimated results would suffer from serious endogeneity problem.

$$Happiness_i = \theta_0 + \theta_1 Exercise_i + \boldsymbol{x_i'\phi} + d_y + d_p + \tau_i \qquad (1)$$

In this model, $Exercise_i$ and $Happiness_i$ are exercise frequency and happiness of respondent i, respectively. $\boldsymbol{x_i'}$ is a vector of control variables. d_y and d_p are time and provincial fixed effects. The main reason for the endogeneity problem in this estimation is reverse causality that happier people may be more willing to participate in physical exercise (Trudel-Fitzgerald et al., 2019; Saunders et al., 2018; Steptoe et al., 2015). This leads τ_i to be correlated with $Exercise_i$, causing a biased estimator of θ_1. Therefore, bi-directionality is one of the sources of endogeneity which means that the explanatory variable is correlated with the error term. If prospective or longitudinal data are available, then the following Fixed Effect (FE) model can be constructed:

$$Happiness_{it} = \delta_0 + \delta_1 Exercise_{it} + \boldsymbol{x_{it}'\varphi} + d_y + d_p + \mu_i + \sigma_{it} \qquad (2)$$

We can use the differencing estimator so as to eliminate the disturbance factor μ_i, which only change with individuals but not with time. The unobserved information contained in the remaining disturbance σ_{it} is much less than that in τ_i, and thus the estimator of δ_1 would be more precise than that of θ_1. This is the reason why prospective and longitudinal datasets are advantageous to tackle endogeneity than cross-sectional data from a statistical point of view.

However, since only a cross-sectional dataset is available to us, this paper utilizes the instrumental variable approach for the casual inference. Specifically, the following two-stage least squares (2SLS) statistical model is constructed.

$$Exercise_i = \alpha_0 + \alpha_1 Automation_i + \boldsymbol{x_i'\psi^1} + d_y + d_p + \varepsilon_i^1 \qquad (3)$$

$$Happiness_i = \beta_0 + \beta_1 \widehat{Exercise_i} + \boldsymbol{x}_i'\boldsymbol{\psi}^2 + d_y + d_p + \varepsilon_i^2 \qquad (4)$$

The instrumental variable used in this paper is the degree to which automation affects the respondents, denoted as $Automation_i$. The first equation is the first stage regression of 2SLS, in which $Automation_i$ is utilized to estimate $Exercise_i$. In the second equation, the predicted values of exercise frequency from the first stage estimation are used to examine its effects on happiness. $Automation_i$ is the instrumental variable constructed by Mihaylov and Tijden (2019), which characterizes the degree of replacement by automation for occupations based on the routine intensity of the tasks at work. According to Angrist and Pischke (2009, chapter 4), Greene (2017, chapter 8) and Wooldridge (2019, chapter 15), if the instrumental variable $Automation_i$ satisfies the following two conditions, $\widehat{\beta_1}$ will be able to unbiasedly estimate the effect of $Exercise_i$ on $Happiness_i$, so as to obtain conclusions of causality. The first requirement is relevance, which means that the instrumental variable $Automation_i$ should have an impact on $Exercise_i$. The second prerequisite is more important because it is the key condition for resolving reverse causality: $Automation_i$ should be exogenous to individual choices, especially not affected by $Happiness_i$. If exogeneity is satisfied, it would mean that the effect of $Happiness_i$ on physical exercise is not included in $\widehat{Exercise_i}$, and consequently $\widehat{Exercise_i}$ is irrelevant to ε_i^2. Therefore, $\widehat{\beta_1}$ would be able to portray the unidirectional impact of physical activity on happiness and thus the reverse causality could be addressed to a large extent.

Theoretically, the instrumental variable used in this paper satisfies these two conditions. Studies have shown that, the higher the degree of replacement by automation, the less time people need to work, and the more time they could spend on recreational activities and physical exercise (Acemoglu and Restrepo, 2020). Therefore, this instrumental variable satisfies the correlation prerequisite. In addition, the impact of automation on occupation is determined by exogenous technological progress and thus is independent of individuals' choices. Consequently, this instrumental variable is exogenous to the estimation equation of happiness levels and unaffected by people's subjective well-being.

Fortunately, we can statistically test whether the instrumental variable meets these two conditions. Table 1 shows the relevant statistical results. Column (1) displays results of the first stage estimation of 2SLS, showing that the effect of $Automation_i$ on $Exercise_i$ is significantly positive at the 1%

level. This supports above theoretical analysis on the correlation condition that the higher the degree of replacement by automation, the more often they could participate in physical exercise. In addition, the F-value of this regression analysis is 28.619, which is much greater than the rule of thumb of 10. It is another strong evidence that the instrumental variable satisfies the correlation condition (Angrist and Pischke, 2009; Greene, 2017; Wooldridge, 2019). To test exogeneity of the instrument variable, referring to the approach of Conley et al. (2012), we perform the following regression:

$$\widehat{\tau_i} = \gamma_0 + \gamma_1 Automation_i + \boldsymbol{x}_i'\boldsymbol{\vartheta} + d_y + d_p + \omega_i \quad (5)$$

Model (5) is a regression of the residuals $\widehat{\tau_i}$ in model (1) on the instrumental variable $Automation_i$. If γ_1 is estimated to be not significant, it would prove that $Automation_i$ is not correlated with $\widehat{\tau_i}$ and thus independent of the random disturbance term in the estimation equation of $Happiness_i$. Results in column (2) of Table 1 show that $Automation_i$ is truly unrelated with $\widehat{\tau_i}$ and therefore the exogeneity condition is proven to be satisfied, meaning that this instrumental variable does help to solve the endogeneity problem of reverse causality. Hence, applying the instrumental variable method, this paper can measure the causal effect of exercise participation on happiness based on dealing with endogeneity to some extent.

Validity test of the instrumental variable

**Model**	**(1)<br>OLS**	**(2)<br>OLS**
**Variable**	**Exercise frequency**	**Residuals in model (1)**
Automation	0.388^{***}<br>(0.117)	-0.001<br>(0.003)
Controls	Yes	Yes
Constant	0.754^{**}<br>(0.370)	0.005<br>(0.269)
Observations	11725	11725

Notes: ***, **, and * indicate significance at the levels of 1%, 5%, and 10%, respectively. The values in parentheses are standard errors robust to heteroskedasticity. Yes means the corresponding variables are controlled in the regression.

Admittedly, the instrument variable approach often falls short of the "gold standard" of randomized controlled trials, because the assignment to the treatment of the instrument variable may not be totally random. In addition, we will be able to better address the endogeneity of reverse causality if we have an ideal prospective sample and apply the instrumental variable method with it.

References:

[1] Acemoglu D, Restrepo P. Robots and jobs: Evidence from US labor markets. J Polit Econ. (2020) 128:2188–244. doi: 10.1086/705716

[2] Angrist J D, Pischke J-S. (2009). Mostly Harmless Econometrics: An Empiricist's Companion. Princeton: Princeton University Press.

[3] Conley T G, Hansen C B, Rossi P E. Plausibly Exogenous. The Review of Economics and Statistics, (2012) 94(1): 260-272. doi: 10.1162/REST_a_00139

[4] Greene W H. (2017). Econometric Analysis (eighth edition). New York: Pearson Education.

[5] Mihaylov E, Tijdens KG.Measuring the routine and non-routine task content of 427 four-digit ISCO-08 occupations. In: Tinbergen Institute Discussion Paper. Amsterdam: Tinbergen Institute (2019) TI 2019-035/V. doi: 10.2139/ssrn.3389681

[6] Saunders C, Huta V, Sweet SN. Physical activity, well-being, and the basic psychological needs: adopting the SDT model of eudaimonia in a post-cardiac rehabilitation sample. Appl Psychol Health Well Being. (2018) 10(3):347-367. doi: 10.1111/aphw.12136

[7] Steptoe A, Deaton A, Stone AA. Subjective well-being, health, and ageing. Lancet. (2015) 385(9968):640-648. doi: 10.1016/S0140-6736(13)61489-0

[8] Trudel-Fitzgerald C, James P, Kim ES, Zevon ES, Grodstein F, Kubzansky LD. Prospective associations of happiness and optimism with lifestyle over up to two decades. Prev Med. (2019) 126:105754. doi: 10.1016/j.ypmed.2019.105754

[9] Wooldridge J M. (2019). Introductory Econometrics: A Modern Approach (seventh edition). Boston: Cengage Learning.

Explanation of the Mechanism Analysis

The mechanism analysis in this paper is based on the three-stage procedure proposed by Baron and Kenny (1). Meanwhile, in view of the endogeneity problem and referring to Alesina and Zhuravskaya (2), a mechanism framework integrated with the two-stage least square regression (2SLS) is applied to investigate the impact mechanism of exercise participation based on dealing with endogeneity. The approach of Alesina and Zhuravskaya (2) used in this paper is fundamentally consistent with that of Baron and Kenny (1), aiming to perform the mechanism analysis while dealing with the problem of endogeneity. The original method of Baron and Kenny (1) is:

$$Happiness_i = \alpha_0 + \alpha_1 \widehat{Exercise_i} + \boldsymbol{x_i'\psi^1} + d_y + d_p + \varepsilon_i^1 \quad (1)$$

$$Mediator_i = \beta_0 + \beta_1 \widehat{Exercise_i} + \boldsymbol{x_i'\psi^2} + d_y + d_p + \varepsilon_i^2 \quad (2)$$

$$Happiness_i = \gamma_0 + \gamma_1 \widehat{Exercise_i} + \gamma_2 Mediator_i + \boldsymbol{x_i'\psi^3} + d_y + d_p + \varepsilon_i^3 \quad (3)$$

The statistical framework proposed by Alesina and Zhuravskaya (2) based on Baron and Kenny's method integrating 2SLS to deal with endogeneity is as follows.

$$Exercise_i = \delta_0 + \delta_1 Automation_i + \boldsymbol{x_i'\psi^4} + d_y + d_p + \varepsilon_i^4 \quad (4)$$

$$Happiness_i = \theta_0 + \theta_1 \widehat{Exercise_i} + \boldsymbol{x_i'\psi^5} + d_y + d_p + \varepsilon_i^5 \quad (5)$$

$$Mediator_i = \vartheta_0 + \vartheta_1 \widehat{Exercise_i} + \boldsymbol{x_i'\psi^6} + d_y + d_p + \varepsilon_i^6 \quad (6)$$

$$Happiness_i = \rho_0 + \rho_1 \widehat{Exercise_i} + \rho_2 Mediator_i + \boldsymbol{x_i'\psi^7} + d_y + d_p + \varepsilon_i^7 \quad (7)$$

$Exercise_i$, $Happiness_i$, $Automation_i$ and $Mediator_i$ represent the exercise participation, happiness, instrumental variable and the mediating variable respectively. $\boldsymbol{x_i'}$ is a vector of a series of control variables. d_p and d_y are the provincial and year fixed effect respectively. Model (4) uses the instrumental variable of $Automation_i$ to estimate $Exercise_i$. In models (5)-(7), the regression is conducted to examine the effect of predicted values of $Exercise_i$. Hence, under the basic framework of Baron and Kenny (1) and meanwhile applying the instrumental variable method, this paper can investigate the mechanisms through which exercise participation impacts happiness based on dealing with endogeneity. If both ϑ_1 and ρ_2 are estimated to be significant, it would prove that $Mediator_i$ plays a mediating role in the effect of exercise participation on subjective well-being. We have added above explanations of this approach to the Supplementary Material.

Furthermore, to check the robustness of findings using the approach of Alesina and Zhuravskaya (2), we also carry out the mechanism analysis based on the original Baron and Kenny (1)'s procedure. Results are demonstrated in the following Table 3, showing consistent findings with that presented in this paper. We add this table to the Supplementary Material.

Mechanism analysis using Baron and Kenny (1)'s original procedure

**Model**	**(1) OLS**	**(2) OLS**	**(3) OLS**	**(4) OLS**	**(5) OLS**	**(6) OLS**	**(7) OLS**
**Variable**	**Happiness**	**Health status**	**Happiness**	**Suffering from health problems**	**Happiness**	**Depression**	**Happiness**
Exercise frequency	0.025*** (0.002)	0.032*** (0.002)	0.019*** (0.002)	-0.035*** (0.002)	0.020*** (0.002)	-0.025*** (0.002)	0.019*** (0.002)
Health status			0.186*** (0.006)				
Suffering from health problems					-0.145*** (0.006)		
Depression							-0.242*** (0.006)
Constant	4.173*** (0.059)	5.018*** (0.069)	3.240*** (0.064)	0.922*** (0.070)	4.308*** (0.059)	1.496*** (0.069)	4.540*** (0.057)
Controls	Yes	Yes	Yes	Yes	Yes	Yes	Yes
Observations	23345	23336	23336	23314	23314	23306	23306

Notes: ***, **, and * indicate significance at the levels of 1%, 5%, and 10%, respectively. The values in parentheses are standard errors robust to heteroskedasticity. Yes means the corresponding variables are controlled in the regression, while No means not controlled. OLS refers to the Ordinary Least Squares model. Column (1) is the estimates using the instrumental variable approach in above analysis, demonstrating the effect of exercise frequency on happiness. Columns (2), (4), (6) present the estimations of exercise participation on mediating variables of physical health status, the frequency of suffering from health problems and depression respectively. Meanwhile, when both the explanatory and mediating variables are included in the regressions, corresponding results are shown in columns (3), (5), (7). The variable of Happiness is from the question in CGSS "Do you feel that you are generally happy in your life?". The answers to this question measure happiness on a five-point Likert-scale with "1-very unhappy", "2-relatively unhappy", "3-cannot say happy or unhappy", "4-relatively happy" and "5-very happy".
